# Physiology of body lateralization on regional lung ventilation and lung volumes in healthy subjects: Within-subjects design

**DOI:** 10.1371/journal.pone.0335622

**Published:** 2025-10-30

**Authors:** Layane S. P. Costa, Cyda M. A. Reinaux, Emanuel F. F. Silva Júnior, Wagner S. Leite, Daniella C. Brandão, Armèle Dornelas de Andrade, Rollin Roldán, Caio C. A. Morais, Shirley Lima Campos

**Affiliations:** 1 Department of Physiotherapy, Federal University of Pernambuco, Recife, Pernambuco, Brazil; 2 Health-Applied Biology Graduate Program at the Federal University of Pernambuco, Recife, Pernambuco, Brazil; 3 Rehabilitation Sciences Doctoral Program, University of Florida, Florida, United States of America; 4 Laboratory of Experimental Physiology, Faculty of Human Medicine, Universidad de Piura, Lima, Perú; 5 Laboratory of Pulmonology LIM-09, Discipline of Pulmonology, Instituto do Coração (Incor), Hospital das Clínicas da Faculdade de Medicina da Universidade de São Paulo, São Paulo, Brazil; Massachusetts General Hospital Department of Anesthesia Critical Care and Pain Medicine, UNITED STATES OF AMERICA

## Abstract

**Introduction:**

Lateral positioning improves pulmonary mechanics and lung volumes, but its effects in healthy adults remain unclear due to individual variability.

**Objective:**

To analyze the acute effects of lateral body positioning on regional lung ventilation and lung volumes in healthy adults.

**Methods:**

This within-subject study included two protocols: supine and left-lateral position (unilateral) with repeated measures and supine, left, and right-lateral positions (bilateral). All positions were performed at 30° for 5 minutes on an automated rotation bed. Electrical Impedance Tomography measured regional lung ventilation (%) and end-expiratory lung volumes (EELV) across four lung regions: (anterior right [AR] and left [AL]; posterior right [PR], and left [PL]). Linear mixed models assessed the influence of body position and individual variability on regional ventilation and lung volumes, while the Restricted Maximum Likelihood method compared between right- and left-lateral positioning.

**Results:**

In the unilateral protocol (n = 29; 58.6% male; 22.8 ± 4.0 years), left-lateral positioning decreased regional ventilation in nondependent regions (AR: −0.96%, PR: −1.63%) and increased it in dependent regions (PL: 1.17%, AL: 1.42%) versus supine (p < 0.001). EELV increased in PL (+ 0.7 mL/kg PBW), PR (+2.0), and AR (+2.8), but decreased in AL (−2.3) (*p* < 0.001). In the bilateral protocol (n = 10, 70% male; 23.6 ± 3.2 years), regional ventilation showed no significant effects of position, ROI, or interaction (*p* > 0.05). However, EELV varied significantly with body position (*p* < 0.001), with no isolated ROI effect (*p* = 1.000).

**Conclusions:**

Lateral positioning improves regional ventilation in dependent lung regions and increases EELV in nondependent and posterior dependent lung regions, regardless of side.

**Trial registration:**

ClinicalTrials.gov [NCT06044896]

## 1. Introduction

Body positioning interventions have been used in clinical practice to optimize lung function in patients with unilateral and bilateral disorders under spontaneous breathing and in critically ill patients on mechanical ventilation [1,2].

Two main lateral positioning techniques have been described. Firstly, traditional lateral positioning refers to placing the body at a 90° angle to the horizontal plane of the bed, causing the gravitational vector to act perpendicular to the lateral axis of the thorax, creating differences in transpulmonary pressure between the dependent (lower) and non-dependent (upper) lungs. This position can cause significant mechanical compression of the dependent lung due to the weight of the mediastinal and abdominal structures, with differences being observed between the right- and left-lateral positions [2,3].

Studies have shown that in the right-lateral position, with the right lung in a gravity-dependent position, the weight of the liver and mediastinum exerts greater compression on the right lung, potentially increasing the ventilation-perfusion (V/Q) mismatch [4]. In contrast, in the left-lateral position, with the right lung in a non-dependent position, the mediastinal impact is distributed more evenly, and the absence of hepatic compression on the right diaphragm allows for greater diaphragmatic excursion and left lung ventilation [5].

Manual static body lateral positioning involves a partial body rotation along its longitudinal axis while maintaining a specific angle. In this context, the gravitational vector acts obliquely on the thorax, causing a less pronounced pressure gradient resulting in a more gradual distribution of gravitational forces throughout the lung parenchyma. This effect reduces mechanical compression of the dependent lung, maintaining a more balanced pressure gradient, which promotes better ventilation distribution [6]. Nowadays, the use of an automated rotation bed allows precise control of the lateralization angle, reducing anatomical interference and variability related to manual positioning [7].

Advances in lateral body positioning and monitoring technologies highlight the need for a better understanding of their physiological effects in healthy individuals. Despite anatomical differences, these individuals exhibit mechanical properties and positional responses sufficiently similar to support generalized physiological conclusions [8]. The gravitational principle dictates a predictable and consistent lung ventilation distribution pattern, regardless of which lung is positioned upwards, reflecting the “functional symmetry” of the right and left lungs. Consequently, ventilation distribution and end-expiratory lung volume (EELV) are expected to be relatively symmetrical between both hemithoraces, reinforcing the concept of functional lung symmetry under normal conditions [9].

This study aimed to assess the acute effects of 30° lateral positioning on the regional distribution of pulmonary ventilation and EELV compared with the supine position in healthy individuals. The initial design adopted a unilateral protocol (supine and left-lateral position), defined because this posture favors diaphragmatic mobility and right lung ventilation, minimizes EIT signal interference, and was assumed to represent contralateral lung behavior. Based on the premise that right- and left-lateral effects are comparable, a subsequent bilateral protocol directly compared both lateral positions. Accordingly, two research questions were addressed: (1) whether 30° left-lateral positioning acutely alters regional ventilation and EELV compared with supine, and (2) whether the effects on regional ventilation and EELV differ between right- and left-lateral positions. The findings may help characterize physiological responses and guide refinement of intervention protocols before clinical application to improve efficacy and safety.

## 2. Methods

### 2.1 Design

This within-subjects study exposed all subjects to each experimental condition, following the recommendations of the Template for Intervention Description and Replication (TIDieR checklist) [10] (S1 Appendix). The study protocol complied with the Declaration of Helsinki and was approved by the Institutional Research Ethics Committee (Protocol No. 5.980.254). The trial was prospectively registered at *ClinicalTrials.gov* (NCT06044896), and written informed consent was obtained from all subjects prior to enrollment. The study was conducted in the clinical simulation laboratory of a public university hospital between September and October 2023.

### 2.2 Participants and eligibility criteria

Healthy adults aged 18–59 years, both sexes, were recruited through consecutive sampling. Exclusion criteria included: body mass index (BMI) >29.9 kg/m^2^; history of smoking; evidence of impaired pulmonary function, defined as values below predicted for pulmonary function tests, respiratory muscle strength (S2 Appendix); and the presence of any absolute or relative contraindications to performing forced respiratory maneuvers during breathing assessments [11,12].

### 2.3 Experimental protocol

Before the study, subjects were informed of all protocol steps and instructed to promptly report any discomfort to allow immediate interruption of the procedure if necessary. It used a Multicare® bed (LINET, Dordrecht, Netherlands), capable of lateralizing to both sides at a 30° angle, and subjects were instructed to remain still while breathing was recorded in supine and lateral positions.

A preliminary transition from supine to a 30° lateral tilt was performed over 1 minute to assess bed cushion positioning, tolerance to positional change (including respiratory and hemodynamic responses), and any potential displacement of the electrical impedance tomography (EIT) belt, which continuously monitored regional ventilation distribution. Vital signs and any signs of discomfort were monitored throughout the experiment.

Eligible subjects were evaluated using a unilateral or bilateral protocol. Between position changes, a 2-minute washout period was implemented to ensure the absence of residual effects. The washout duration was determined through a pilot study by three respiratory care experts (L.S., C.M., and S.C.). Continuous monitoring of the global plethysmogram and EELV derived from EIT demonstrated that this interval was sufficient for immediate stabilization of regional ventilation distribution and EELV across all regions of interest (ROIs) during transitions between supine and 30° positions in both directions.

**Fig 1:**
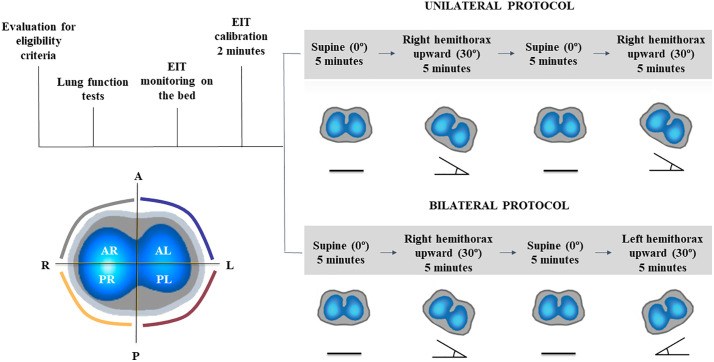
Experimental protocol for analyzing the effects of 30° unilateral and bilateral lateral positioning in healthy individuals monitored by EIT. Legend: The study included an initial eligibility assessment, including pulmonary function tests, followed by baseline EIT monitoring and calibration in the supine position. Subjects underwent two positioning protocols: (1) Unilateral protocol – two repeated positioning sequences alternating between the supine (0°) and left lateral positions (right hemithorax upward, 30° tilt) for 5 minutes in each position, following the sequence: supine–left lateral–supine–left lateral; and (2) Bilateral protocol – a sequence alternating between the supine (0°), left-lateral, and right-lateral positions for 5 minutes in each position, following the sequence: supine–left lateral–supine–right lateral. Transverse EIT images illustrate the regional distribution of lung volume across quadrants. Abbreviations: AR, anterior right; AL, anterior left; PR, posterior right; PL, posterior left.

In the unilateral protocol, subjects were monitored in the supine position (reference) and in a 30° left-lateral tilt, with the right hemithorax placed in the non-dependent position. This sequence was repeated twice, with each position maintained for 5 minutes. For the bilateral protocol, a different group of subjects followed the sequence: supine, 30° tilt with the right hemithorax in the non-dependent position, washout, supine, and 30° tilt with the left hemithorax in the non-dependent position (Fig 1).

### 2.4 Data collection

For data acquisition, an EIT monitor (Enlight 1800, Timpel, Brazil) was connected to a 32 electrodes-belt. Correct belt placement was verified through four sequential steps: (1) anatomical positioning by manual palpation to ensure placement between the 4th and 5th intercostal spaces along the mid-axillary line [13], with the belt positioned perpendicular to the ground at a 90° angle; (2) verification of EIT signal quality following a stabilization period of at least 1 minute; (3) independent validation by two investigators to confirm electrode placement in an orthogonal plane; and (4) functional confirmation of adequate ventilation distribution in dynamic images was performed according to consensus recommendations through a transition test from supine to 30° left- and right-lateral inclination (three repetitions), evaluating electrode adherence and symmetry of tidal ventilation patterns [14].

These procedures were implemented to prevent rotation or tilting of the measurement plane, which could result in inaccurate estimates of ventilated areas if positioned too high, or interference from diaphragmatic movement if positioned too low. Additionally, the quality of electrode-skin contact was verified [15]. During lateral positioning, belt height was maintained constant relative to anatomical landmarks, preventing craniocaudal shifts in the measurement plane. The risk of displacement or rotation was minimized through the use of fixed anatomical references, absence of direct manual contact with subjects, and body stabilization to the bed using cushions, ensuring minimal likelihood of measurement artifacts [14].

Global tidal volume was measured using a flow sensor (Respironics Novametrix, Wallingford, Connecticut, USA) connected to an inflatable cushion mask. Plethysmography calibration for each subject included measurement of 10 baseline breaths followed by three repeated forced inspiration maneuvers. This calibration determined the end-expiratory lung impedance (EELI) in mL, a surrogate for EELV [13,14]. All data were recorded in a single continuous file with timestamps for each positional change. For analysis, recordings were segmented into supine and lateral frames, with repeated measurements for each 5-minute period in the respective positions.

### 2.5 Outcomes

The primary outcomes were changes in regional ventilation distribution (ΔZ) and changes in EELV. Lung ventilation distribution was expressed as a percentage, calculated as:

$\begin{array}{c} Ventilation\;ROI\;(\%)\;=\;\left[\frac{\Delta 𝐙\;(roi)}{\Delta 𝐙\;(global)}\right]\;\times 100 \end{array}$

Where ΔZ (ROI) and ΔZ (Global) represent impedance variations in the regions of interest and in the global plethysmogram, respectively. Changes in EELV were scaled from arbitrary units (AU) to milliliters (mL) using the average slope (scaling factor) derived from respiratory cycles simultaneously monitored with a flow sensor prior to the interventions [15,16]. EIT data were converted and processed using Timpel S.A. software (EIT Analysis, version 1.6.1).

For interindividual comparison, EELV values were normalized to predicted body weight (PBW) according to ARDSNet [17] reference equations: for males, PBW (kg) = 50 + 0.91 × (height [cm] – 152.4); for females: PBW (kg) = 45.5 + 0.91 × (height [cm] – 152.4). Normalized EELV was expressed in mL/kg PBW.

ΔZ and EELV were analyzed for ROIs defined by including only pixels with regional impedance variation (ΔZ) exceeding 10% of the maximum value observed in the image, representing at least 10% of relative ventilation compared to the pixel with the highest ΔZ. To compare the changes in lung regional ventilation distribution and regional EELV between positions, the ROIs were: anterior right (AR), anterior left (AL), posterior right (PR), and posterior left (PL). This quadrant segmentation allows simultaneous evaluation of physiological variations along the anteroposterior and lateral axes, providing adequate spatial resolution to distinguish between dependent and non-dependent lung regions, as well as to detect differences between the right and left hemithorax, allowing the identification of regional asymmetries associated with anatomical or functional factors [18].

### 2.6 Sample size

Sample size calculation was performed using an online calculator specifically designed for within-subjects studies [19], based on preliminary data obtained from a pilot study involving ten subjects. The variables ΔZ (%) and EELV, the latter expressed in milliliters per kilogram of predicted body weight (mL/kg of PBW) were analyzed across four lung quadrants: AR, AL, PR, and PL in both supine and lateral positions. No superiority was observed between variables regarding estimated sample size requirements.

Thus, an initial sample size of 13 subjects was estimated as sufficient for this two-treatment design, ensuring 80% power to detect a difference at a two-sided significance level of 0.05. All calculations were normalized to PBW (S1 Table). However, considering the individual variability observed in the pilot study, a larger sample was ultimately collected.

### 2.7 Statistical analysis

Descriptive statistics included mean (standard deviation), median (interquartile range), or counts and percentages, as appropriate. Normality of distribution was analysed by the Shapiro–Wilk test.

Differences in regional lung ventilation distribution and EELV mL/kg of PBW between supine and lateral positions were analyzed using a linear mixed model for repeated measurements, with Position, ROIs, and their interaction included as fixed effects, and a random intercept for subjects to account for within-subject correlation. Parameter estimation was performed with 95% confidence intervals (CI) and corresponding p-values.

Data from bilateral protocol were analyzed using the Restricted Maximum Likelihood method. The significance of main and interaction effects was assessed using the Wald test (Omnibus), reporting χ^2^ values, degrees of freedom, and p-values. When significant effects were detected, paired post hoc comparisons were performed using estimated marginal means, adjusted with the Bonferroni method for multiple testing (S4 Appendix). All analyses were performed in R (RStudio, version 4.3.1; R Foundation for Statistical Computing, Vienna, Austria) with statistical significance set at p < 0.05.

## 3. Results

**Fig 2:**
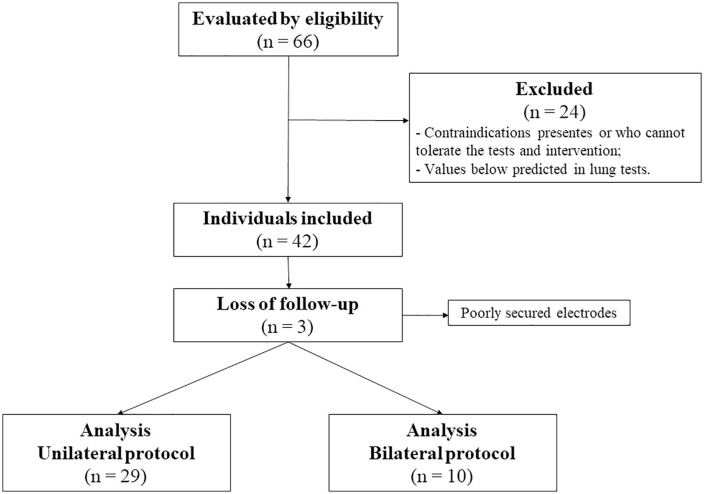
Flowchart of study.

Sixty-six subjects were assessed for eligibility. Of these, twenty-four were excluded due to values below the expected range in pulmonary function and respiratory muscle strength tests and/or alterations in respiratory patterns (S3 Appendix) (S2 Table). Additionally, three subjects were excluded from the analysis due to signal noise in the EIT, possibly caused by electrode detachment during position changes (Fig 2). A total of 39 subjects were enrolled: 29 in the unilateral protocol (58.6% male; 22.8 ± 4.0 years) and 10 in the bilateral protocol (70% male; 23.6 ± 3.2 years). Subjects were predominantly young, physically active, with median height and weight within the normal range. Pulmonary function tests were within the reference values established by the American Thoracic Society/European Respiratory Society [12], and respiratory parameters were within normal limits (Table 1).

**Table 1:** Characteristics of the study subjects (N = 39).

Demographic data	Unilateral protocol (N = 29) Mean ± SD N (%)	Bilateral protocol (N = 10) Mean ± SD N (%)
Age (years)	22.8 ± 4.0	23.6 ± 3.2
Sex (male)	17 (58.6)	7 (70)
Height (cm)	168.4 ± 8.0	172.2 ± 9.4
Weight (kg)	68.6 ± 13.6	71.9 ± 21.9
Body mass index (kg/m^2^)	24.0 ± 3.6	22.8 ± 3.5
Physical activity (yes)	19 (65)	6 (60)
Chest circumference (cm)	91.1 ± 9.0	88.6 ± 7.2
Abdominal circumference (cm)	80.0 ± 9.3	81.0 ± 10.4
**Vital signs at rest**		
Oxygen saturation (%)	98.1 ± 1.0	98.3 ± 0.5
Heart rate (beats/minute)	80.6 ± 15.8	78.0 ± 8.0
Respiratory Rate (breaths/minute)	16.5 ± 3.1	17.2 ± 2.5
Systolic blood pressure (mmHg)	115.0 ± 10.0	118.0 ± 6.3
Diastolic blood pressure (mmHg)	77.5 ± 8.0	80.0 ± 6.6
**Respiratory muscle strength**		
Maximal inspiratory pressure (cmH_2_O)	110.4 ± 33.8	106.6 ± 31.6
Maximal expiratory pressure (cmH_2_O)	115.7 ± 27.8	120.1 ± 31.8
**Lung function**		
FVC (L)	4.2 ± 0.8	4.7 ± 0.8
FVC (%, predicted)	97.7 ± 14.2	97.3 ± 11.3
FEV_1_ (L)	3.5 ± 0.7	3.8 ± 0.6
FEV_1_ (%, predicted)	93.2 ± 13.8	92.7 ± 9.5

FVC (L) – Forced Vital Capacity in liters; FVC (%, predicted) – Forced Vital Capacity expressed as a percentage of the predicted; FEV_1_ (L) – Forced Expiratory Volume in 1 second, in liters; FEV_1_ (%, predicted) - Forced Expiratory Volume in 1 second expressed as a percentage of the predicted; L – liters; mL - milliliter; kg – kilogram(s); kg/m^2^ - kilogram(s) per square meter; cm – centimeter(s); mmHg - millimeters of mercury; cmH_2_O - centimeters of water.

**Fig 3:**
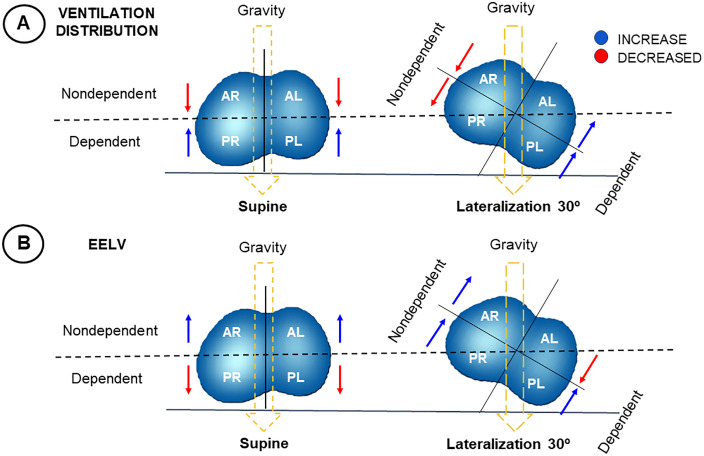
Effects of changes in ventilation distribution and EELV in the supine and 30° left-lateral positioning. The two panels refer to the ventilation distribution (A) and EELV (B) monitored by EIT, comparing the effects of changes in the supine (0°) and lateral (30°) positions, emphasizing the influence of gravity. Panel A – Ventilation distribution: In the supine position, ventilation is reduced in the nondependent quadrants (AR, AL) and higher in the dependent quadrants (PR, PL) lung regions. In the 30° left-lateral, gravity (yellow dashed line) shifts the dependent (lower) lung regions downward, leading to decreased ventilation (red arrows) in nondependent areas (AR, PR) and increased ventilation (blue arrows) in dependent areas (AL, PL). Panel B – EELV: In the supine position, dependent regions tend to have lower aeration due to gravitational effects. In the 30° left-lateral, only the upper dependent region (AL) shows a reduction in EELV. Abbreviations: AR – right anterior; AL – left anterior; PR – right posterior; PL – left posterior.

The analysis of the variation in ventilation distribution (Fig 3A) and EELV (Fig 3B) was illustrated through a thoracic cross-sectional view of the four quadrants (AR, AL, PR, and PL), both in the supine and left-lateral inclined to a 30°. The arrows indicate the gravitational action and the changes during the transition from the supine to the 30° left-lateral position, aiding in the understanding of the physiological behavior.

**Fig 4:**
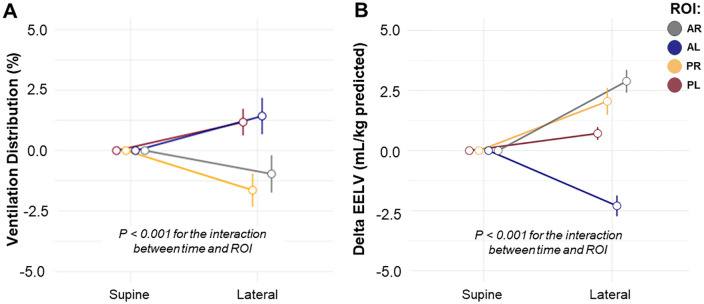
Variation of regional ventilation and end-expiratory lung volume (EELV) distribution resulting from positional changes in healthy individuals. **A –** EELV mL/kg of PBW for interaction between position, inter-ROIs and sequence of intervention; **B –** Percentage of regional ventilation distribution for interaction between position, ROIs and sequence of intervention; **Abbreviations:** AR, Anterior Right; AL, Anterior Left; PR, Posterior Right; PL, Posterior Left; PBW, Predicted Body Weight.

Comparisons of regional ventilation distribution between supine and left-lateral position (right hemithorax positioned upwards with an inclination of 30°) showed a decrease in the ventilation distribution of both ROIs of the nondependent lung by −0.96% (95% CI −1.71 to −0.21) and −1.63% (95% CI −2.29 to −0.97) for AR and PR, respectively, while the ventilation distribution increased in both ROIs of the dependent lung by 1.17% (95% CI 0.65 to 1.69) and 1.42% (95% CI 0.69 to 2.15) for PL and AL, respectively. Changes in regional ventilation distribution due to positional changes were statistically significant in all the ROIs analyzed (p < 0.001 for interaction between body positions and ROIs) (Fig 4A). Considering within-ROI ventilation distribution, significant changes from supine to lateral position were observed in all four ROIs, with p < 0.001 for the AL, PR, PL, and p = 0.007 for AR (S1 Fig).

Otherwise, comparisons of regional EELV between supine and left-lateral position showed an increase in EELV of both ROIs of the nondependent lung by 2.8 mL/kg of PBW (95% CI 0.22 to 0.66, p < 0.001) and 2 mL/kg of PBW (95% CI 0.27 to 0.79, p < 0.001) for AR and PR, respectively; while in the dependent lung, EELV increased in the PL by 0.7 mL/kg of PBW (95% CI 0.12 to 0.36, p < 0.001) and decreased in the AL by −2.3 mL/kg of PBW (95% CI 0.21 to 0.61, p < 0.001). The interaction between the supine to lateral positional change and the four ROIs with repeated measures was significant for EELV mL/kg of PBW (p < 0.001) (Fig 4B).

**Fig 5:**
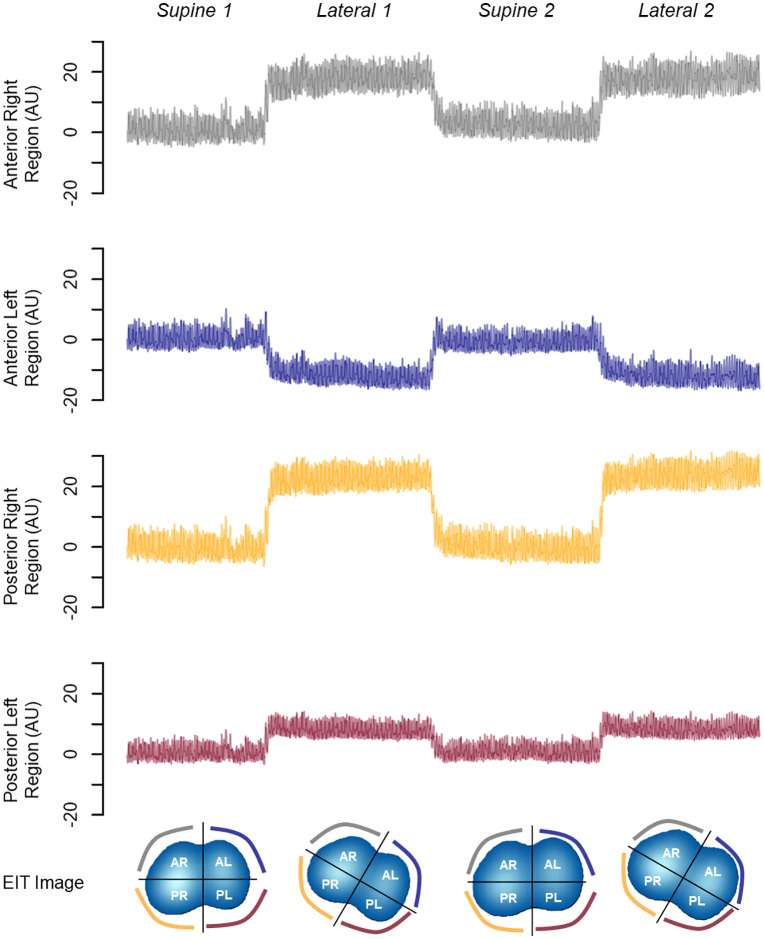
Representation of plethysmography curves for the ROIs anterior right (AR), anterior left (AL), posterior right (PR), posterior left (PL), and functional electrical impedance tomography (EIT) images, representing ventilatory changes from 30° left-lateral position to supine in a case from the study.

Our results were consistent, as the physiological effects on the ROIs through EIT plethysmography were also observed when analyzing the returning from the left-lateral position to the supine position with the same magnitudes of change (Fig 5).

In the bilateral protocol, no significant differences were observed in regional ventilation distribution between left- and right-lateral inclinations (p > 0.05) (S2 Fig). For EELV (mL/kg of PBW), a significant effect of Position was found (χ^2^(12) = 421.19; p < 0.001), with no effect of ROI (χ^2^(3) = 0.00; p = 1.000) (S3 Fig). Post hoc comparisons revealed that the most pronounced differences occurred between the lateral positions. In the AL quadrant, the left-lateral position showed a marked increase compared to the right-lateral position (+7.37; *padj* = 0.003). Conversely, in the AR quadrant, a significant reduction was observed in the left-lateral position compared to the right-lateral position (−6.54; *padj* < 0.001) (S4 Appendix).

Additional differences were observed in specific quadrants: in PL, an increase in the left-lateral compared to the right-lateral (+1.72; *padj* = 0.014) and in the supine position compared to the left (+0.70; *padj* = 0.008) and right-lateral (+2.42; *padj* = 0.005); in PR, the supine position was higher than the left-lateral (+1.54; *padj* = 0.009); in AL, a decrease in supine versus left-lateral (−2.61; **padj* *= 0.002) and an increase versus right-lateral (+4.76; *padj* = 0.005); and in AR, an increase in supine compared to left-lateral (+3.60; *padj* = 0.001) and a decrease compared to right-lateral (−2.95; *padj* = 0.001).

## 4. Discussion

The principal findings of this study, conducted in healthy volunteers, were as follow: (1) In the unilateral protocol, repositioning from supine position to a 30° lateral tilt with the right hemithorax in the non-dependent position redistributed ventilation, increasing it in the dependent regions and reducing it in non-dependent ROIs. This was accompanied by an increase in EELV in both ROIs of the non-dependent lung, whereas the dependent lung showed opposite changes, with an increase in the left posterior ROI and a decrease in the left anterior ROI; (2) In the bilateral protocol, regional ventilation distribution did not differ significantly between the right- and left-lateral positions; however, EELV changes were position-dependent, with lateral tilts producing opposite effects in the anterior lung regions according to the side positioned upward, indicating a redistribution of lung volume.

Normal pulmonary ventilation is regionally heterogeneous, with greater ventilation occurring in dependent than in non-dependent lung areas due to a gravitational ventilation gradient [6]. A 30° lateral tilt modifies the gas-tissue ratio, particularly in the basal lung regions, where gravitational effects are most pronounced, promoting a more homogeneous pattern than upright or supine positions [20]. In this posture, the gravitational pressure gradient also favors ventilation of dependent lung regions that are typically more susceptible to collapse, reducing alveolar ventilation heterogeneity and improving ventilation in these regions [21]. Consistent with these physiological principles, our results showed a greater distribution of ventilation in the dependent lung and reduced ventilation in non-dependent regions during lateral positioning. This pattern reflects gravitational redistribution combined with the thoracic restriction imposed by the supporting surface, which reduces chest wall compliance, aligning with previous studies in healthy subjects [20].

The increase in non-dependent EELV was expected, reflecting higher transpulmonary pressure secondary to reduced regional pleural pressure [8,22]. In both protocols, a 30° left-lateral tilt with the right hemithorax non-dependent produced opposite EELV changes in the dependent lung quadrants, with a decrease in the left anterior (AL) and an increase in the left posterior (PL), whereas in the bilateral protocol, a 30° right-lateral tilt with the left hemithorax non-dependent caused a reduction exclusively in the right anterior quadrant (AR), a finding consistent with the expected pattern [22]. Possible mechanisms could explain this increase in EELV in the dependent quadrants, including an increase in the lateral axis diameter relative to the anteroposterior, restriction of thoracic expansion leading to better ventilation distribution of the dependent lung, and the influence of abdominal pressure gradients on transpulmonary pressures in the lateral position [23]. Cranial displacement of the diaphragm with forward abdominal contents, further amplifies this phenomenon [9].

This study initially focused on the left-lateral position, placing the right side in the non-dependent position, supported by previous evidence indicating minimal differences between lateral positions regarding physiological, anatomical, and mechanical breathing properties [22]. The right-lung–upward was considered more favorable position for investigating the effects of lateralization in this population because the impact of cardiac compression on the left lung is minimized compared with the influence of the liver on the diaphragm, thereby favoring greater diaphragmatic mobility, enhancing right-lung ventilation, and promoting more homogeneous ventilation with a lower ventilation/perfusion (V/Q) mismatch [24]. This lateral positioning also balances mediastinal weight, improving pulmonary compliance and gas exchange.

The bilateral protocol isolated the effects of lateralization on pulmonary ventilation and demonstrated symmetric redistribution of ventilation during lateral positioning, with no significant differences between left and right inclinations. In healthy subjects, left–right symmetry of the ventilatory pattern during body lateralization was expected given preserved lung and chest wall mechanics, consistent with previous EIT findings in spontaneously breathing subjects [25]. Accordingly, no significant differences were observed in regional ventilation distribution or global EELV between left- and right-lateral inclinations. Small but significant differences in EELV (increase in AL and decrease in AR during left-lateral inclination) likely reflect transitional changes between gravity-dependent and non-dependent regions and subtle anatomical and mechanical asymmetries, including the influence of the heart and mediastinum on the dependent lung, without compromising overall ventilatory symmetry. These findings underscore gravity-driven redistribution of ventilation and end-expiratory volume with postural change and emphasize the need to control belt height and body alignment to reduce measurement bias [25,26].

Similar changes in EELV with 30° lateral body positioning have been reported in bilateral protocols by Roldán et al. (2022) [22] in patients with COVID-19-related acute respiratory distress syndrome (ARDS), by Mlček et al. (2023) [27] in an animal model of ARDS, and by Huerta et al. (2025) [28] in mechanically ventilated patients. Mlček et al. (2021) [21] using EIT analyzed a “directed” lateral positioning strategy with personalized bedside PEEP, aiming to selectively attenuate alveolar overdistension and collapse in patients with COVID-19-associated ARDS and right-left pulmonary aeration/ventilation asymmetry. Lateral positioning can not only prevent pulmonary collapse but also minimize overdistension, thereby reducing ventilation heterogeneity, which may represent a clinical benefit as relevant as the prevention of pulmonary collapse itself.

This study has important limitations. Although EIT is a valuable bedside tool, it cannot quantify absolute lung volumes, including EELV, because measurements rely on relative impedance changes influenced by factors unrelated to ventilation (e.g., perfusion, cardiac activity, diaphragmatic motion, fluid accumulation). Chest geometry, body positioning, and electrode placement may also create artifacts and reduce spatial resolution. Although these factors were carefully controlled, they must be considered when interpreting EIT data. The duration of each body position was guided by plethysmogram feedback. Finally, the absence of esophageal pressure measurements and diaphragmatic ultrasound limited additional insights.

These findings may support future studies aimed at translating the physiological understanding of unilateral and bilateral lateralization into clinically applicable protocols for different patient populations, and at clarifying whether similar response patterns occur in unilateral or bilateral pulmonary impairment. Prospective studies combining EIT-derived metrics with physiological assessments, such as respiratory mechanics and diaphragmatic excursion, may help determine the optimal degree and duration of lateral tilts and evaluate their effects on gas exchange, regional aeration, and clinical outcomes. Continuous real-time monitoring of the effects of lateral positioning through EIT opens new perspectives for personalized clinical applications and dynamic therapeutic adjustments based on ΔZ, EELV, and the pattern of morphofunctional impairment [14]. These advances may bridge the gap between experimental research and clinical practice, promoting the use of lateral positioning as a physiology-guided intervention in various respiratory care settings.

## 5. Conclusion

In healthy subjects, the transition from the supine position to a 30° lateral position, with either the right or left hemithorax in the nondependent position, results in greater ventilation distribution in the dependent lung, accompanied by an increase in EELV in the nondependent lung and in the posterior quadrant of the dependent lung. These findings were observed in both unilateral protocols, which assessed exclusively the lateral decubitus on one side, and bilateral protocols, which alternately considered both sides, reinforcing the consistency of the effects of lateralization on regional ventilation distribution and EELV.

## Supporting information

S1 Appendix Template for Intervention Description and Replication Checklist (TIDieR). (DOCX)

S2 Appendix Respiratory assessments. (DOCX)

S3 Appendix Exclusion Criteria of the Subjects. (DOCX)

S4 Appendix Bilateral Protocol. (DOCX)

S1 Table Values obtained in the Sample Calculation. (DOCX)

S2 Table Exclusion criteria of study subjects. (DOCX)

S1 Fig Ventilation distribution in intra-ROIs, with the individual response of each case can be observed in the unilateral protocol when positioned in the left lateral posture from the supine position. (DOCX)

S2 Fig Changes in ventilation distribution across different lung positions and regions of interest (ROIs), assessed by electrical impedance tomography (EIT), in a bilateral protocol. The graph shows variations in regional ventilation distribution at four distinct time points: supine, left-lateral, supine and right-lateral. Measurements were obtained in four regions of interest (ROIs): right anterior (AR), left anterior (AL), right posterior (PR), and left posterior (PL).
(DOCX)

S3 Fig Changes in EELV (mL/kg of PBW) across different lung positions and regions of interest (ROIs), assessed by electrical impedance tomography (EIT), in a bilateral protocol. The graph shows variations in EELV, adjusted for predicted body weight, at four distinct time points: supine, left-lateral, supine and right-lateral. Measurements were obtained in four regions of interest (ROIs): right anterior (AR), left anterior (AL), right posterior (PR), and left posterior (PL).
(DOCX)
