# Supplementary material for: Physiology of body lateralization on regional lung ventilation and lung volumes in healthy subjects: Within-subjects design
Source: PLoS One. 2025 Oct 30;20(10):e0335622. doi: 10.1371/journal.pone.0335622 (PMC12574891; doi:10.1371/journal.pone.0335622)
Supplement: S2 Appendix — (DOCX) [file pone.0335622.s002.docx]

**S2 Appendix.** Respiratory assessments

Pulmonary function was assessed using the EasyOne Plus Diagnostic® spirometer (Andover, USA), measuring forced vital capacity (FVC) and the volume expired in the first second (FEV_1_). Respiratory muscle strength was estimated by measuring maximal inspiratory and expiratory pressures using the digital manometer MVD300-U® (São Paulo, Brazil). The individuals were instructed to perform the respiratory maneuvers according to the recommendations of the American Thoracic Society/European Respiratory Society (2019), and the predicted values were calculated based on references for the Brazilian population [1-3].

**REFERENCES**

1[. ATS/ERS Statement on Respiratory Muscle Testing. Am J Respir Crit Care Med. 2002;166: 518–624. doi:10.1164/rccm.166.4.518](https://www.zotero.org/google-docs/?broken=LgdOFL)

2[. Pessoa IMBS, Houri Neto M, Montemezzo D, Silva LAM, Andrade ADD, Parreira VF. Predictive equations for respiratory muscle strength according to international and Brazilian guidelines. Braz J Phys Ther. 2014;18: 410–418. doi:10.1590/bjpt-rbf.2014.0044](https://www.zotero.org/google-docs/?broken=Wl0JkD)

3. Graham BL, Steenbruggen I, Miller MR, Barjaktarevic IZ, Cooper BG, Hall GL, et al. Standardization of Spirometry 2019 Update. An Official American Thoracic Society and European Respiratory Society Technical Statement. Am J Respir Crit Care Med 2019 ;200(8):e70–88. . doi/10.1164/rccm.201908-1590ST
