# Supplementary material for: Physiology of body lateralization on regional lung ventilation and lung volumes in healthy subjects: Within-subjects design
Source: PLoS One. 2025 Oct 30;20(10):e0335622. doi: 10.1371/journal.pone.0335622 (PMC12574891; doi:10.1371/journal.pone.0335622)
Supplement: S3 Appendix — (DOCX) [file pone.0335622.s003.docx]

**S3 Appendix -** Exclusion Criteria of the Subjects

Most of the volunteers were excluded due to pulmonary function tests below the predicted values (FVC and FEV_1_). However, other criteria were also considered, such as muscle strength below the predicted value and the irreproducibility of maneuver values (differences greater than 10% between repetitions). All pulmonary function test sessions were reproducible, conducted by specialists, and participants repeated the tests when altered results were found. Although the recruitment was aimed at healthy participants with clear inclusion criteria, the volunteers reported being unaware of any respiratory health condition, even after we explained the concept of a "healthy lung" and the smoking history (none of the participants were smokers) (S2 Table).
