# Supplementary material for: Physiology of body lateralization on regional lung ventilation and lung volumes in healthy subjects: Within-subjects design
Source: PLoS One. 2025 Oct 30;20(10):e0335622. doi: 10.1371/journal.pone.0335622 (PMC12574891; doi:10.1371/journal.pone.0335622)
Supplement: S4 Appendix — (DOCX) [file pone.0335622.s004.docx]

**S4 Appendix.** Bilateral Protocol

Based on the hypothesis that the effects of the right and left lateral positions are comparable, a subsequent bilateral protocol was conducted to evaluate both lateral positions. Within this context, two research questions were addressed: (1) whether the left lateral position at 30° induces acute changes in regional ventilation and EELV compared to the supine position; and (2) whether the effects on regional ventilation and EELV differ between the right and left lateral positions. Ten individuals (70% male; 23.6 ± 3.2 years) were evaluated following the protocol: supine, left lateral, washout, supine, and right lateral. This analysis also aimed to characterize lateralization patterns on both sides, providing additional support for the physiological interpretation of the main findings.

Data were analyzed using linear mixed models to comprehensively assess the effects of position, regions of interest (ROIs), and the position × ROI interaction on two main physiological variables: ventilation distribution (S2 Fig) and changes in end-expiratory lung volume normalized to predicted body weight (delta EELV mL/kg of PBW) (S3 Fig).

To represent postural transitions, the position variable was coded as: supine, left lateral, supine, right lateral. The ROIs corresponded to the lung quadrants AR, AL, PR, and PL, previously defined by EIT, allowing spatial analysis of ventilation. The intercept for each participant was modeled as a random effect to account for within-subject correlation arising from repeated measurements across different positions and regions. Model parameters were estimated using the restricted maximum likelihood method. The overall significance of each effect was assessed using the Wald (omnibus) test, with reporting of chi-square (χ²) values, corresponding degrees of freedom, and *p*-values."

To investigate specific differences, post hoc comparisons were performed within each ROI for three predefined contrasts: supine vs. left lateral, supine vs. right lateral, and left lateral vs. right lateral. These comparisons were conducted with Bonferroni adjustment for multiple testing. For each contrast, the mean differences (Position B – Position A), 95% confidence intervals (95% CI), *t*-values, degrees of freedom (df), and both raw and adjusted *p*-values were reported. A significance level of 5% (*p* < 0.05) was adopted based on the adjusted *p*-values.

In the analysis of ventilation distribution, no significant effects of position, ROI, or the position × ROI interaction were observed (*p* > 0.05 for all). In contrast, for delta EELV (mL/kg PBW), a significant effect of position was identified (χ²(12) = 421.19; *p* < 0.001), with no isolated effect of ROI (χ²(3) = 0.00; *p* = 1.000). Post hoc comparisons revealed that the most pronounced differences occurred between the lateral positions. In the AL quadrant, the left lateral position showed a marked increase compared with the right lateral (+7.37; 95% CI: 3.91 to 10.84; *padj* = 0.003). Conversely, in the AR quadrant, a significant reduction was observed in the left lateral compared with the right lateral (−6.54; 95% CI: −9.02 to −4.06; *padj* < 0.001).
