# Supplementary material for: Physiology of body lateralization on regional lung ventilation and lung volumes in healthy subjects: Within-subjects design
Source: PLoS One. 2025 Oct 30;20(10):e0335622. doi: 10.1371/journal.pone.0335622 (PMC12574891; doi:10.1371/journal.pone.0335622)
Supplement: S1 Table — (DOCX) [file pone.0335622.s005.docx]

**S1 Table.** Values obtained in the Sample Calculation

| **Variables** | **SSD** | **MDD** | **N** |
| --- | --- | --- | --- |
| EELV AR (mL) | 149 | 92.58 | 23 |
| EELV AL (mL) | 127 | 78.74 | 23 |
| EELV PR (mL) | 107 | 66.52 | 23 |
| EELV PL (mL) | 39.75 | 24.39 | 23 |
| EELV AR (mL/PBW) | 1.44 | 1.24 | 13 |
| EELV AL (mL/PBW) | 1.20 | 1.02 | 13 |
| EELV PR (mL/PBW) | 1.56 | 1.35 | 13 |
| EELV PL (mL/PBW) | 0.98 | 0.85 | 13 |
| Delta Z AR (%) | 3.01 | 2.63 | 13 |
| Delta Z AL (%) | 3.48 | 3.04 | 13 |
| Delta Z PR (%) | 2.33 | 2.02 | 13 |
| Delta Z PL (%) | 1.75 | 1.52 | 13 |

**Note:** **SDD** - Standard deviation of the difference between the two values for the same patient; **MDD** - The smallest difference between the treatments or strength of association that you wish to be able to detect; mL - milliliter; PBW - predicted body weight; AR - anterior right; AL - anterior left; PR - posterior right; PL - posterior left.
