# Supplementary material for: Physiology of body lateralization on regional lung ventilation and lung volumes in healthy subjects: Within-subjects design
Source: PLoS One. 2025 Oct 30;20(10):e0335622. doi: 10.1371/journal.pone.0335622 (PMC12574891; doi:10.1371/journal.pone.0335622)
Supplement: S2 Table — (DOCX) [file pone.0335622.s006.docx]

**S2 Table.** Exclusion criteria of study subjects

| **VOLUNTEERS** | **EXCLUSION CRITERIA** | **POSSIBLE CAUSES** |
| --- | --- | --- |
| Volunteer 7 | FEV_1_ 54% of the predicted and FVC 71% of the predicted | Sedentarism |
| Volunteer 8 | FEV_1_ 61% of the predicted | Sedentarism |
| Volunteer 17 | FEV_1_ 65% of the predicted | Sedentarism / Asthma as a child / History of COVID |
| Volunteer 21 | FEV_1_ 69% of the predicted and FVC 72% | History of COVID-19 |
| Volunteer 22 | MIP 66,5% of the predicted | Sedentarism |
| Volunteer 24 | FVC 55% of the predicted | Meningitis as a child / Sedentarism |
| Volunteer 26 | FEV_1_ 73% of the predicted | Not identified |
| Volunteer 27 | FEV_1_ 79% of the predicted | Not identified |
| Volunteer 29 | FEV_1_ 41% of the predicted and FVC 70% of the predicted | History of COVID-19 |
| Volunteer 30 | Non-reproducible values of respiratory muscle strength (above 10% difference between repetitions) | The participant reported a history of asthma |
| Volunteer 33 | FEV_1_ 80% of the predicted and FVC 77% of the predicted | History of COVID-19 |
| Volunteer 36 | FEV_1_ 72% of the predicted and FVC 77% of the predicted | Not identified |
| Volunteer 38 | FVC 77% of the predicted | Asthma as a child |
| Volunteer 40 | FVC 76% of the predicted | Not identified |
| Volunteer 41 | MIP 65% of the predicted | Not identified |
| Volunteer 43 | Non-reproducible values of respiratory muscle strength (above 10% difference between repetitions) | Not identified |
| Volunteer 44 | FVC 71% of the predicted | History of COVID-19 |
| Volunteer 47 | FEV_1_ 75% of the predicted | Sedentarism |
| Volunteer 49 | FVC 70% of the predicted | Asthma as a child |
| Volunteer 50 | FEV_1_ 71% of the predicted | Sedentarism |
| Volunteer 52 | FEV_1_ 75% of the predicted and FVC 75% of the predicted | History of hypertensive peaks |
| Volunteer 53 | FEV_1_ 73% of the predicted | History of COVID-19 |
| Volunteer 55 | Non-reproducible values of respiratory muscle strength (above 10% difference between repetitions) | Not identified |
| Volunteer 56 | FEV_1_ 78% of the predicted | Sedentarism |

**Abbreviations:** FEV_1_ - Forced Expiratory Volume in the first second; FVC - Forced Vital Capacity; MIP - Maximum Inspiratory Pressure.
