# Supplementary material for: Physiology of body lateralization on regional lung ventilation and lung volumes in healthy subjects: Within-subjects design
Source: PLoS One. 2025 Oct 30;20(10):e0335622. doi: 10.1371/journal.pone.0335622 (PMC12574891; doi:10.1371/journal.pone.0335622)
Supplement: S1 Fig — (DOCX) [file pone.0335622.s007.docx]

**
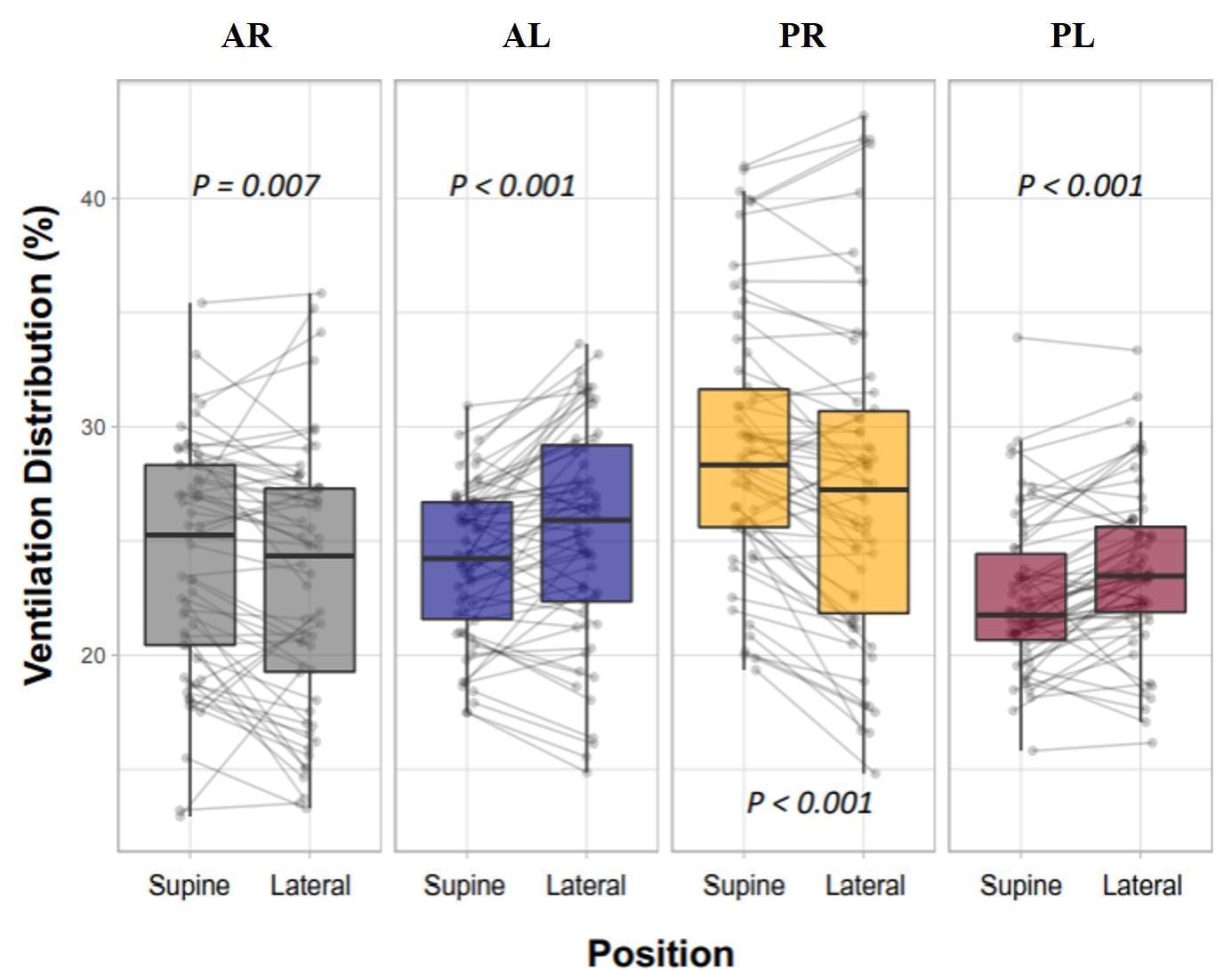
**

**S1 Fig.** Ventilation distribution in intra-ROIs, with the individual response of each case can be observed in the unilateral protocol when positioned in the left lateral posture from the supine position.
