# Supplementary material for: Physiology of body lateralization on regional lung ventilation and lung volumes in healthy subjects: Within-subjects design
Source: PLoS One. 2025 Oct 30;20(10):e0335622. doi: 10.1371/journal.pone.0335622 (PMC12574891; doi:10.1371/journal.pone.0335622)
Supplement: S2 Fig — The graph shows variations in regional ventilation distribution at four distinct time points: supine, left-lateral, supine and right-lateral. Measurements were obtained in four regions of interest (ROIs): right anterior (AR), left anterior (AL), right posterior (PR), and left posterior (PL). (DOCX) [file pone.0335622.s008.docx]

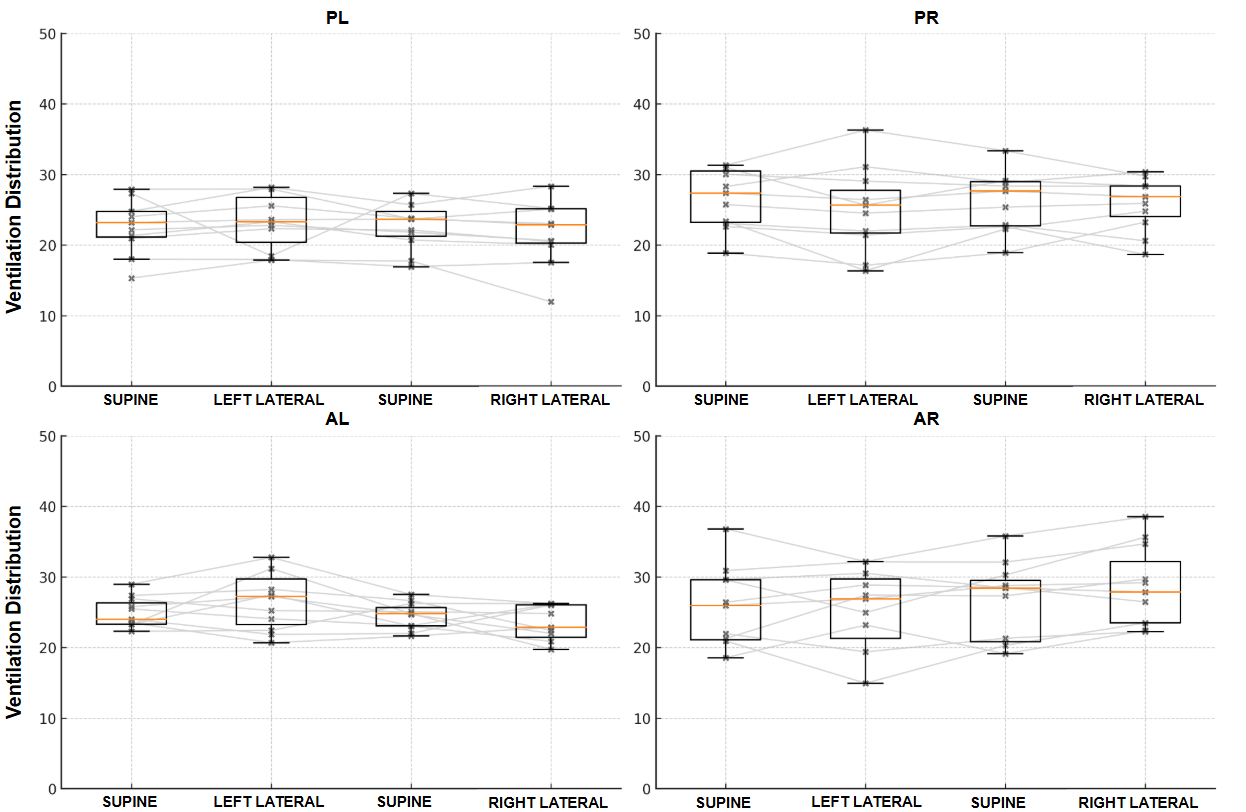


**S2 Fig.** Changes in ventilation distribution across different lung positions and regions of interest (ROIs), assessed by electrical impedance tomography (EIT), in a bilateral protocol. The graph shows variations in regional ventilation distribution at four distinct time points: supine, left-lateral, supine and right-lateral. Measurements were obtained in four regions of interest (ROIs): right anterior (AR), left anterior (AL), right posterior (PR), and left posterior (PL).
